# Concurrent stunting and overweight or obesity among under-five children in sub-Saharan Africa: a multilevel analysis

**DOI:** 10.1186/s13690-023-01138-8

**Published:** 2023-06-30

**Authors:** Melkamu Aderajew Zemene, Denekew Tenaw Anley, Natnael Atnafu Gebeyehu, Getachew Asmare Adella, Gizachew Ambaw Kassie, Misganaw Asmamaw Mengstie, Mohammed Abdu Seid, Endeshaw Chekol Abebe, Molalegn Mesele Gesese, Natnael Amare Tesfa, Yenealem Solomon Kebede, Berihun Bantie, Sefineh Fenta Feleke, Tadesse Asmamaw Dejenie, Wubet Alebachew Bayeh, Anteneh Mengist Dessie

**Affiliations:** 1 Department of Public Health, College of Health Sciences, Debre Tabor University, Debre Tabor, Ethiopia; 2 Department of Midwifery, College of Medicine and Health Science, Wolaita Sodo University, Wolaita Sodo, Ethiopia; 3 Department of Reproductive Health and Nutrition, School of Public Health, Woliata Sodo University, Wolaita Sodo, Ethiopia; 4 Department of Epidemiology and Biostatistics, School of Public Health, Woliata Sodo University, Wolaita Sodo, Ethiopia; 5 Department of Biochemistry, College of Health Sciences, Debre Tabor University, Debre Tabor, Ethiopia; 6 Unit of Physiology, Department of Biomedical Science, College of Health Science, Debre Tabor University, Debre Tabor, Ethiopia; 7 School of Medicine, College of Health Science, Woldia University, Woldia, Ethiopia; 8 Department of Medical Laboratory Science, College of Health Sciences, Debre Tabor University, Debre Tabor, Ethiopia; 9 Department of Comprehensive Nursing, College of Health Sciences, Debre Tabor University, Debre Tabor, Ethiopia; 10 Department of Public Health, College of Health Sciences, Woldia University, Woldia, Ethiopia; 11 Department of Medical Biochemistry, College of Medicine and Health Sciences, University of Gondar, Gondar, Ethiopia; 12 Department of Maternal and neonatal health Nursing, College of Health Sciences, Debre Tabor University, Debre Tabor, Ethiopia; 13 Department of Epidemiology and preventive Medicine, School of Public Health and Preventive Medicine, Faculty of Medicine, Nursing and Health Sciences, Monash University, Melbourne, Victoria Australia

**Keywords:** Concurrent stunting and overweight or obesity, The double burden of malnutrition

## Abstract

**Background:**

Globally, the co-occurrence of stunting and overweight or obesity (CSO) in the same individual is becoming an emerging layer of malnutrition and there is a paucity of information in low- and middle-income countries, particularly in sub-Saharan Africa. Hence, this study aimed to determine the pooled prevalence and determinants of concurrent stunting and overweight or obesity among under-five children in SSA.

**Methods:**

Secondary data analysis was conducted from a recent nationally representative Demographic and Health Survey dataset of 35 SSA countries. A total weighted sample of 210,565 under-five children was included in the study. A multivariable multilevel mixed effect model was employed to identify the determinant of the prevalence of under-5 CSO. The Intra-class Correlation Coefficient (ICC) and Likelihood Ratio (LR) test were used to assess the presence of the clustering effect. A p-value of *p < 0.05* was used to declare statistical significance.

**Result:**

The pooled prevalence of concurrent stunting and overweight/obesity among under-five children was 1.82% (95% CI: 1.76, 1.87) in SSA. Across the SSA regions, the highest prevalence of CSO was reported in Southern Africa (2.64%, 95% CI: 2.17, 3.17) followed by the Central Africa region (2.21%, 95% CI: 2.06, 2.37). Under five children aged 12–23 months (AOR = 0.45, 95% CI: 0.34, 0.59), 24–35 months (AOR = 0.41, 95% CI: 0.32, 0.52), 36–59 months (AOR = 055, 95% CI: 0.43, 0.70), ever had no vaccination (AOR = 1.25, 95% CI: 1.09, 1.54), under-five children born from 25 to 34 years mother (AOR = 0.75, 95% CI: 0.61, 0.91), under-five children born from overweight/obese mothers (AOR = 1.63, 95% CI: 1.14, 2.34), and under-five children living in West Africa (AOR = 0.77, 95% CI: 0.61, 0.96) were significant determinants for under-five CSO.

**Conclusion:**

Concurrent stunting and overweight or obesity is becoming an emerging layer of malnutrition. Under five children born in the SSA region had almost a 2% overall risk of developing CSO. Age of the children, vaccination status, maternal age, maternal obesity, and region of SSA were significantly associated with under-five CSO. Therefore, nutrition policies and programs should base on the identified factors and promote a quality and nutritious diet to limit the risk of developing CSO in early life.

**Table:** 

Text box 1. Contributions to the literature
• The co-existence of two different forms of malnutrition is known as the double burden of malnutrition and could occur at country, household, or individual level.
• At individual level, stunting might couple with consumption of high energy dense foods that results in a clustering of nutritional problems such as concurrence of stunting and overweight/obesity.
• This study can contribute to filling gaps in the literature regarding the prevalence and determinants of CSO, an emerging layer of malnutrition at individual level. This can help to identify specific risk factors and inform targeted interventions to address this complex issue.

## Background

A lack of optimal nutrition is referred to as malnutrition and can result from either an intake of insufficient nutrients and/or energy (undernutrition) or an intake of excessive nutrients and/or energy (overnutrition) [1]. Both undernutrition and overnutrition have different effects on the child’s growth, development, and cognitive performance. Child malnutrition hurts a person’s ability to survive, develop physically and cognitively, reproduce, and productive capacity. It also makes people more susceptible to develop acute and chronic illnesses later during adulthood period [2]. Furthermore, childhood undernutrition is also strongly correlated with adult overweight and obesity. Undernourished children who were born underweight or stunted are at a much-increased risk of becoming overweight and obese when exposed to energy-dense meals and a sedentary lifestyle later in life [3].

The double burden of malnutrition (DBM) is “*characterized by the coexistence of undernutrition (stunting) along with overweight/obesity, and may lead to diet-related non-communicable diseases (NCD) within individuals, households, and populations across the life course*” [4]. The double burden of malnutrition occurred at the household level when at least one member in the household may be undernourished (i.e., stunted, wasted, or underweight) and at least one member is overweight/obese [5]. It is also defined at the individual level as; an individual is stunted during early life and may be overweight later in life; or an individual may have a co-existence of micronutrient deficiencies with overweight or obesity at the same time [5]. Therefore, a child may suffer from both forms of malnutrition, the co-existence of stunting and overweight or obesity (CSO) at the same time [6–9].

Globally, about 162 million children under the age of five are thought to be stunted [10]. Of these, around 90% of the global burden of stunting occurs in 36 African and Asian countries [11]. Recent studies in Africa evidenced that the proportion of children who are stunted ranges from 18.8 to 46% [12–14] with an average prevalence of 41% [15]. Early growth stunting remains a serious public health issue, and it is strongly linked to poor cognitive function and academic performance [16].

In many parts of the world, the prevalence of obesity among children is rising at an alarming rate. However, little attention has been paid to the more recent rise of overweight and obesity in low- and middle-income countries [17]. The pooled prevalence of overweight and/or obesity in sub-Saharan Africa was 5.10% with a higher prevalence in the Southern region (8.8%) [18]. Childhood overnutrition is associated with obesity-related medical conditions like cardiovascular diseases, liver disease, type 2 diabetes mellitus, asthma, and other respiratory diseases. Moreover, childhood obesity has also a negative impact on a child’s physical health, and social and emotional well-being including low self-esteem and depression [19].

Few studies investigated the prevalence of concurrence stunting and overweight or obesity in the same individual. The magnitude of co-occurrence of stunting and overweight or obesity among under-five children in Ghana was 1.2% [7], Ethiopia 1.99% [6], Kenya 1.1% [20], Vietnam 2.7% in 2013 and 1.4% in 2016 [9], 5% and 10% among indigenous and non-indigenous Mexican children respectively [21]. Previous studies evidenced that breastfeeding status, child’s age, maternal education, maternal age, maternal height, wealth status, and family size were identified as factors that had an association with concurrent stunting and overweight or obesity among children [6, 7, 9, 20–23].

The co-existence of stunting and overweight or obesity (CSO) in the same children is a new layer of malnutrition and there is a paucity of information in low- and middle-income countries, particularly in the sub-Saharan region. The prevalence of CSO was relatively small in previous studies (< 10%), indicating using a pooled dataset increases the statistical power to identify any statistical difference and gives the researchers and policymakers a clearer understanding of the burden of the problem in sub-Saharan Africa. Therefore, this study aimed to determine the pooled prevalence and determinants of concurrent stunting and overweight or obesity (CSO) among under-five children in sub-Saharan Africa using nationally representative Demographic and Health Survey datasets.

## Methods and materials

### Data source, study area, and period

The data for this study was extracted from the recent Demographic Health Survey (2010–2021) datasets of 35 sub-Saharan African countries. Sub-Saharan Africa contains countries from four geographical regions namely; Central Africa, East Africa, Southern Africa, and West Africa. The data were accessed from the DHS program’s official database www.measuredhs.com after permission was granted through an online request.

### Study design and sampling procedures

Demographic Health Survey is a nationally representative population-based cross-sectional study design. It uses a similar sampling methodology and data collection procedures across the countries. DHS used a two-stage stratified cluster sampling technique. In the first stage, a sample of EAs was selected independently from each stratum with proportional allocation stratified by residence (urban & rural). In the second stage, from the selected EAs, households were taken by systematic sampling technique. Then, from the selected households, measurements of weight and height were taken from children aged 0–59 months. Recumbent length measurements were taken of children under the age of 24 months while standing height measurements were taken of if they were older than 24 months. Weight measurements were taken on lightweight SECA mother-infant scales with digital displays that were developed and produced by UNICEF. Detailed sampling and data collection procedures were also available from the full DHS report [12].

### Study population and samples

The source population was all under-five children in the five years preceding each respective survey in sub-Saharan Africa, whereas those in the selected Enumeration Areas (EAs) were the study population. The sample size was determined from the kids to recode file “KR file” datasets with at least one survey from 2010 to 2021. Children who were not weighed and measured and children whose values for weight and height were not recorded are excluded. For anthropometry indices that use age in the computation, children whose months or years of birth are missing or ambiguous were excluded. A total sample size of 212,479 (weighted 210,565) under-five children were included in this study.

### Variables

The outcome variable was a concurrence of stunting and overweight/obesity (CSO) within the same individual. CSO was taken as a binary response; 1 coded “Yes” for the co-existence of stunting and overweight/obesity and 0 coded for “No”. Stunting was defined as a height-for-age Z-score (HAZ) below − 2SD and overweight/obesity was defined as a weight-for-height (or length) z-score (WHZ) above 2 SD as compared to the median value of World Health Organization (WHO) 2006 growth standards reference point [24]. The independent variables were thematized as child characteristics, maternal characteristics, household characteristics, and community-level factors.

### Operation definition

#### Concurrent stunting and overweight/obesity (CSO)

Children were classified as CSO if they had a HAZ of < -2SD and their WHZ > + 2SD simultaneously.

#### Wealth index

is a composite measure of a household’s cumulative living standard divided into 5 quantiles using the wealth quantile data derived from the principal component analysis [12].

### Data analysis

The data were coded, verified, and analyzed by using STATA version 16/MP software. The overall analysis in this study was carried out on weighted data to restore representativeness and complex sampling procures were also considered during the testing of statistical significance. Due to the hierarchical nature of the DHS data where child characteristics are nested in the community, multilevel binary logistic regression analysis was employed to identify the factors associated with concurrence stunting and overweight/obesity. Thus, five models were fitted and model comparison was made using deviance information criteria (DIC) for the models were nested models. Null model (empty model), Model I (child characteristics), Model II (Model I + maternal characteristics), Model III (Model II + household characteristics), and Model IV (Model III + community level characteristics) were fitted, and a model with the lowest deviance was chosen as the best-fitted model for the data. The Intra-class Correlation Coefficient (ICC) and Likelihood Ratio (LR) test were used to assess the presence of the clustering effect. The Intra-class Correlation Coefficient (ICC) was the proportion of total variance in the outcome variable that was attributed to the area level.

ICC = VA/(VA + VI), where VA was the area-level variance and VI corresponded to individual-level variance, (VI = π^2^/3 = 3.29).

Variables with a p-value < 0.25 were selected for multivariable analysis. In the multivariable analysis, an Adjusted Odds Ratio (AOR) with a 95% Confidence Interval (CI) was reported and statistical significance was declared at p-value < 0.05.

## Results

### Sociodemographic characteristics and distribution of CSO in SSA

A total weighted sample of 210,565 under-five children who were born in the last five years preceding the survey was included in the study. The median age of children was 27 months with an Inter-Quartile Range (IQR) of 13–43 months. The lowest prevalence of concurrent stunting and overweight/obesity was observed in Gambia at 0.50% (95% CI:0.30, 0.80) and the highest was from South Africa with a prevalence of 5.42% (95% CI: 4.16, 6.95) (Table 1).

**Table 1:** Distribution of concurrent stunting and overweight/obesity in sub-Saharan Africa countries (n = 210,565)

Countries	Study year	Number of under five children	Prevalence of CSO (95% CI)
Angola	2015/16	6003	2.01 (1.67, 2.40)
Benin	2017/18	11,762	0.76 (0.61, 0.93)
Burkina Faso	2010	6994	1.37 (1.11, 1.67)
Burundi	2016/17	6238	0.91 (0.69, 1.18)
Cameron	2018	4760	5.07 (4.45, 5.72)
Chad	2014/15	10,149	1.18 (0.98, 1.41)
Comoros	2012	2982	3.92 (3.25, 4.68)
Congo Republic	2011/12	4009	1.35 (1.01,1.75)
Ivory Coast	2011/12	3132	1.27 (0.92, 1.73)
D. R. Congo	2013/14	8231	2.42 (2.10, 2.78)
Ethiopia	2016	9735	1.76 (1.51, 2.04)
Gabon	2012	3039	2.20 (1.71, 2.79)
Gambia	2019/20	3515	0.50 (0.30, 0.80)
Ghana	2014	2654	0.94 (0.61, 1.38)
Guinea	2018	3439	4.58 (3.91, 5.34)
Kenya	2014	17,499	1.20 (1.04, 1.37)
Lesotho	2014	1344	2.70 (1.88, 3.68)
Liberia	2019/20	2185	1.77 (1.27, 2.43)
Madagascar	2021	5742	1.32 (1.04, 1.65)
Malawi	2015/16	5244	2.27 (1.88, 2.70)
Mali	2018	8934	1.33(1.10, 1.59)
Mauritania	2019-21	10,033	0.57 (0.44, 0.74)
Mozambique	2011	10,426	4.44 (4.05, 4.85)
Namibia	2013	1703	0.84 (0.45, 1.37)
Niger	2012	5830	1.25 (0.98, 1.57)
Nigeria	2018	11,517	1.05 (0.87, 1.25)
Rwanda	2019	3924	2.09 (1.66, 2.58)
Seaira Leone	2013	4102	2.69 (2.21, 3.22)
Senegal	2010/11	4120	1.16 (0.86, 1.54)
South Africa	2016	1085	5.42 (4.16, 6.95)
Tanzania	2015/16	8853	1.87 (1.60, 2.17)
Togo	2013/14	3089	0.74 (0.47, 1.11)
Uganda	2016	4373	1.35 (1.03, 1.73)
Zambia	2018	8668	2.91 (2.57, 3.29)
Zimbabwe	2015	5256	2.56 (2.15, 3.03)

### The pooled prevalence of CSO among under-five children in SSA

**Fig. 1:**
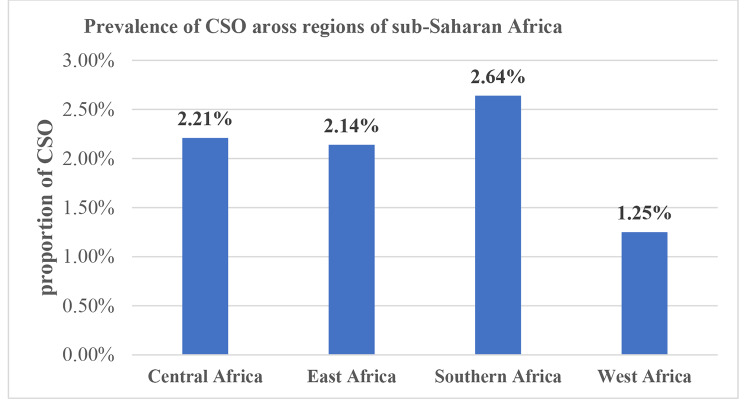
Prevalence of concurrent stunting and overweight/obesity in regions of sub-Saharan African

Overall, the pooled prevalence of concurrent stunting and overweight/obesity among under-five children was 1.82% (95% CI: 1.76, 1.87) in SSA. Across the regions of SSA, the highest prevalence of CSO was from Southern Africa (2.64%, 95% CI: 2.17, 3.17) followed by the Central Africa region (2.21%, 95% CI: 2.06, 2.37). The West Africa region had the lowest prevalence of CSO among under-five children (Fig. 1).

### Prevalence of CSO in children’s characteristics

The magnitude of concurrent stunting and overweight/obesity varied in terms of different characteristics. For instance, the risk of CSO was higher (1.93%) among male children than females (1.71%). Similarly, the co-existence of stunting and overweight/obesity was high in children less than six months (4.71%) followed by 6–11 months (1.76%). Moreover, the prevalence of CSO among under-five children who had not received Vitamin A supplementation was 2.54% as compared to those who had taken it (1.53%) (Table 2).

**Table 2:** Prevalence of concurrent stunting and overweight/obesity by study participant’s characteristics in sub-Saharan Africa

Variables	Categories	Weighted frequency (%)	Prevalence of CSO (%)
Child’s sex	Male	106,374 (50.2)	1.93
	Female	104,191 (49.48)	1.71
Child’s age in months	< 6 months	23,976 (11.39)	4.71
	6–11 months	23,409 (11.12)	1.76
	12–23 months	43,734 (20.77)	1.43
	24–35 months	40,646 (19.30)	1.51
	36–59 months	78,799 (37.42)	1.33
Currently breast feed	No	90, 086 (42.78)	1.46
	Yes	120,478 (57.22)	2.09
Received VA supplementation	No	59,164 (28.10)	2.54
	Yes	151,401 (71.90)	1.53
Ever had vaccination(n = 62,634)	No	13,303 (21.24)	2.78
	Yes	49,331 (78.76)	1.54
Child size at birth	Small	46,597 (22.13)	1.62
	Average	97,259 (46.19)	1.83
	Large	66,709 (31.68)	1.95
Birth type	Single	204,013 (96.89)	1.81
	Twin	6,552 (3.11)	1.98
Maternal age in years	15–24	58,368 (27.72)	2.33
	25–34	102,443 (48.65)	1.64
	35–49	49,754 (23.633)	1.58
Maternal educational status	No education	82,286 (39.08)	1.68
	Primary	73,699 (34.99)	1.96
	Secondary	54,610 (25.93)	1.84
Maternal stature (n = 171,559)	Short	49,530 (28.87)	2.27
	Normal/tall	122,029 (71.13)	1.71
Maternal BMI (n = 170,823)	Underweight	16,197 (9.48)	1.34
	Normal	113,394 (66.38)	1.87
	Overweight/obese	41,232 (24.14)	2.09
Marital status of the mother	In union	15,385 (75.22)	1.75
	Not in union	52,179 (24.78)	2.02
Residence	Urban	66,344 (31.51)	1.75
	Rural	144,220 (68.49)	1.85
Media exposure	No	74,414 (35.34)	1.92
	Yes	136,150 (64.66)	1.76
Wealth index	Poorest	48,122 (22.85)	1.96
	Poorer	45,283 (21.51)	1.83
	Medium	42,336 (20.11)	1.79
	Richer	40,134 (19.06)	1.91
	Richest	34,690 (16.47)	1.53
Family size	≤ 5	85,067 (40.40)	1.97
	> 5	125,498 (59.60)	1.71
Water source	Unimproved	106,007 (50.34)	1.82
	Improved	104, 558 (49.66)	1.81
Toilet facility	Unimproved	127,431 (60.52)	1.84
	Improved	83,134 (39.48)	1.79

### Factors associated with CSO among under-five children

The null model of the multilevel mixed-effect regression analysis revealed that the intraclass correlation coefficient was 5.35%, indicating the presence of a clustering effect. Therefore, fitting a multilevel analysis and controlling a regional-level dependency is mandatory.

In the full models of multivariable mixed effect logistic regression analysis, the age of the children, ever-had vaccination, maternal age, maternal BMI, and regions of sub-Saharan Africa were the independent determinants of concurrent stunting and overweight/obesity among under-five children in SSA. The prevalence of CSO among those under five was 1.82%, which is fair to call the odds effect size as a relative risk. Under five children aged 12–23 months had 55% less risk to develop concurrent stunting and overweight/obesity than children of age less than 12 months (AOR = 0.45, 95% CI: 0.34, 0.59). Similarly, those in the age range of 24–35 months and 36–59 months had 59% (AOR = 0.41, 95% CI: 0.32, 0.52) and 45% (AOR = 055, 95% CI: 0.43, 0.70) less risk to develop CSO than whose age was less than 12 months respectively.

The risk of developing concurrent stunting and overweight/obesity among under-five children who ever had no vaccination was 25% more likely as compared to those who ever had the vaccine (AOR = 1.25, 95% CI: 1.09, 1.54). Under five children who were born between 25 and 34 years old mothers had 25% less risk to develop CSO than their counterparts (AOR = 0.75, 95% CI: 0.61, 0.91). Children who were born from overweight and/or obese mothers had 63% more risk to develop CSO than those who were born from underweight mothers (AOR = 1.63, 95% CI: 1.14, 2.34). Furthermore, under-five children living in West Africa were at 23% lower risk to develop CSO (AOR = 0.77, 95% CI: 0.61, 0.96) (Table 3).

**Table 3:** Factors associated with concurrent stunting and overweight/obesity among under five children in sub-Saharan Africa

Variables	Null model (Model I)	Model I (child characteristics)	Model II (Model I + Maternal characteristics)	Model III (Model II + Household characteristics)	Model IV (Model III + community level characteristics)
Sex of child					
Male		1	1	1	1
Female		0.95 (0.83, 1.11)	0.97 (0.83, 1.13)	096 (0.82, 1.13)	0.96 (0.82, 1.13)
Age of child (months)					
< 12		1	1	1	1
12–23		0.42 (0.33, 0.54)	0.45 (0.35, 0.59)	0.45(0.34, 0.58)	0.45 (0.34, 0.59) **
24–35		0.43 (0.34, 0.53)	0.42 (0.32, 0.54)	0.41 (0.32, 0.53)	0.41 (0.32, 0.52) **
36–59		0.51 (0.41, 0.64)	0.55 (0.43, 0.70)	0.54 (0.43, 0.69)	055 (0.43, 0.70) **
Birth interval					
< 36 months		1	1	1	1
>=36 months		1.03 (0.89, 1.19)	0.97 (0.82, 1.15)	0.99 (0.84, 1.17)	0.98 (0.83, 1.13)
Breast feeding					
No		1	1	1	1
Yes		1.05 (0.87, 1.26)	0.97 (0.79, 1.20)	0.96 (0.78, 1.18)	0.96 (0.78, 1.18)
Child size at birth					
Normal		1	1	1	1
Larger		1.10 (0.91, 1.31)	1.09 (0.90, 0.33)	1.09 (0.90, 1.33)	1.09 (0.90, 1.33)
Small		1.03 (0.83, 1.26)	1.14 (0.90, 1.43)	1.13 (0.90, 1.33)	1.11 (0.88, 1.40)
Ever had vaccine					
Yes		1	1	1	1
No		1.26 (1.02, 1.56)	1.29 (1.03, 1.63)	1.28 (1.02, 1.61)	1.25 (1.09, 1.54) *
Vaccinated for measles					
Yes		1	1	1	1
No		1.04 (0.84, 129)	1.05 (0.83, 1.32)	1.03 (0.82, 1.29)	1.06 (0.84,1.33)
VA supplementation					
Yes		1	1	1	1
No		1.05 (0.89, 1.23)	1.05 (0.88,1.26)	1.04 (0.87, 1.24)	1.03 (0.86, 1.23)
Maternal age					
15–24			1	1	1
25–34			0.76 (0.63, 0.92)	0.76 (0.62, 0.92)	0.75 (0.61, 0.91) **
35–59			0.82 (0.64, 1.03)	0.80 (0.62, 1.02)	0.78 (0.60, 1.03)
Maternal stature					
Normal/tall			1	1	1
Short			1.2 (1.01, 1.45)	1.19 (0.99, 1.42)	1.14 (0.94, 1.34)
Marital status					
In union			1	1	1
Not in union			0.87 (0.70, 1.07)	0.84 (0.68, 1.05)	0.82 (0.66, 1.01)
Maternal BMI					
Underweight			1	1	1
Normal			1.25 (0.94, 1.68)	1.27 (0.95, 1.70)	1.28 (0.95, 1.71)
Overweight/obese			1.49 (1.04, 2.13)	1.60 (1.11, 2.28)	1.63 (1.14, 2.34) **
Maternal educational status					
No education			1	1	1
Primary			1.17 (0.95, 1.43)	1.21 (0.98, 1.47)	1.09 (0.88, 1.33)
Secondary & above			1.22 (0.95, 1.56)	1.38 (1.05, 1.81)	1.29 (0.98, 1.70)
Family size					
≤ 5				1	1
> 5				1.05 (0.88, 1.25)	1.08 (0.90, 1.29)
Media exposure					
No				1	1
Yes				0.93 (0.77, 1.12)	0.96 (0.79, 1.17)
Wealth index					
Poorest				1	1
Poorer				0.87 (0.71, 1.08)	0.88 (0.71, 1.09)
Middle				0.83 (0.65, 1.06)	0.84 (0.66,1.08)
Richer				0.83 (0.64, 1.08)	0.85 (0.65, 1.11)
Richest				0.65 (0.45, 0.94)	0.68 (0.45, 1.02)
Residence					
Urban					1
Rural					1.07 (0.82, 1.40)
Sub-regions of SSA					
Central Africa					1
East Africa					1.19 (0.96, 1.14)
Southern Africa					1.61 (0.92, 2.81)
West Africa					0.77 (0.61, 0.96) *
Model comparation					
ICC (%)	5.35%	10.03%	11.34%	11.3%	10.87%
LL	-19,019	-5389	-4722	-4715	-4700
Deviance	38,038	10,778	9445	9430	9401

VA = vitamin A; BMI = body Mass index; SSA = sub-Saharan Africa; *significant at P < 0.05; **significant at P < 0.001

## Discussion

Poorest low- and middle-income countries are suffering from undernutrition as a result of food insecurity from manmade and/or natural disasters and overnutrition due to nutritional transition, lifestyle change, and the effect of globalization [25]. Hence, the Double burden of malnutrition has become an emerging public health problem in sub-Saharan Africa in the last two decades [26]. Therefore, the current study aimed to determine the pooled prevalence and determinants of concurrence stunting and overweight or obesity among children under the age of five in sub-Saharan Africa.

The pooled prevalence of CSO among under-five children in SSA was 1.82%. The magnitude was slightly higher in males than in females (1.93% vs. 1.71%). The finding from the current study was in line with a study conducted among non-indigenous Guatemalan children of age 0–59 months with a prevalence of 1.9% [27]. However, this finding is lower than a previous study done (4.3%) in the low and middle-income countries of the Middle East and North Africa (MENA) [28], 7.2% in twenty-seven provinces of Indonesia [23]. Besides, this finding is also lower than the result (2.7%) from a study conducted in Vietnam in 2013 [9]. On the other hand, the current study finding was higher than a study reported (0.4%) from a population-based survey in nine cities in China [29], 0.5% in Latin America and the Caribbean (LAC) region [28], and 1.4% in Vietnamese children in 2016 [9]. This difference might be due to socio-economic and socio-cultural differences related to nutrition across regions. Moreover, the cause for this variance could be due to variations in the prevalence of malnutrition between nations or regions, dietary variations, cultural feeding practices, food preferences among children under the age of five, or methodological variations.

This study revealed that older children had a lesser risk of developing CSO than younger children. Under five children aged 12–23 months had 55% less risk to develop CSO than children of age less than 12 months. Similarly, those in the age range of 24–35 months and 36–59 months had 59% and 45% less risk to develop CSO as compared to children whose ages were less than 12 months respectively. This is consistent with previous studies [6, 23] indicating that younger children were more likely to develop concurrent stunting and overweight or obesity as compared to older children. The double burden of malnutrition is high in younger children because they may not be receiving a balanced diet with sufficient nutrients, while also being exposed to unhealthy foods high in fats, sugars, and salt. This can lead to stunted growth and development, as well as an increased risk of obesity and chronic diseases later in life [25, 30]. Younger children are more vulnerable to malnutrition because their bodies are still growing and developing, and they require more nutrients to support their growth [31, 32]. Additionally, younger children may not have access to a diverse range of nutritious foods or may not be able to consume enough food due to poor appetite or illness. However, with proper care, nutrition, and access to healthcare during the first 1000 days of life, malnutrition can be prevented in younger children. Therefore, promoting exclusive breastfeeding for the first six months and scaling up access to nutrient-dense complementary food for young children can be pursued as a medium-term intervention. As a long-term intervention, implementing policies to improve food security and promote sustainable agriculture is recommended.

The vaccination status of the child had associated with the risk of CSO. The risk of developing CSO among under-five children was 25% higher for a child who ever had no vaccination than ever who had the vaccine. This association might be justified as vaccines can prevent infectious diseases that can cause undernutrition. Malnourished children are more vulnerable to infections, which can further exacerbate their nutritional status. By protecting individuals against infectious diseases such as measles, pneumonia, and diarrhea, vaccines can help reduce the burden of undernutrition [33, 34]. On the other hand, vaccines can promote healthy growth and development, which can help prevent overweight and obesity. Childhood obesity is a growing problem in many countries, and it can lead to a range of health issues in adulthood, including diabetes, heart disease, and certain cancers. By preventing infections and promoting healthy growth, vaccines can help reduce the risk of obesity later in life [35]. Thus, improving vaccination rates can be an important component of a comprehensive strategy to reduce CSO, highlighting the complex nature of nutrition.

Under five children who were born between 25 and 34 years old mothers had 25% less risk to develop CSO than their counterparts. This finding was supported by a study conducted in Ghana [7], and Mexico [21] where children who were born from younger women were at higher risk of malnutrition. Children born to younger women are more vulnerable to the double burden of malnutrition [30, 36]. This is because younger women often have poor nutritional status themselves, which can lead to poor fetal growth and development. Additionally, younger mothers may lack the knowledge and resources to provide adequate nutrition for their children, which can further exacerbate the problem. Children who were born from overweight and/or obese mothers had 63% more risk to develop CSO. A similar finding was reported from a Mexican study [21] that concurrent stunting and overweight or obesity was associated with maternal obesity. Factors including biological, behavioral, environmental, socioeconomic, and demographic factors, as well as the recent nutrition transition might play a role in the observed association. The impact of maternal obesity extends beyond intrauterine and neonatal life to childhood, adolescence, and adulthood [37, 38]. According to cohort research, the incidence of obesity among children born to obese mothers was twice as high by the age of two [39]. Furthermore, maternal obesity can lead to changes in the placenta, which can affect nutrient transfer to the fetus. Therefore, while the mother may be obese, the developing fetus may not be receiving adequate nutrition, which can result in a malnourished child. Hence, findings from this study revealed that supportive policies and investments that prioritize younger and obese mothers are significant to reduce the double burden of childhood malnutrition.

In this study, children living in the West African region were at 23% lower risk to develop CSO. This finding is in line with the World Health Organization report that stated there were regional variations in child malnutrition in sub-Saharan Africa [40]. The burden of this new layer of malnutrition, CSO can vary in sub-Saharan African regions due to a variety of factors such as different poverty levels, lack of access to clean water and sanitation, limited access to healthcare, inadequate food supply, and political instability. Besides, differences in cultural practices and dietary habits can also play a role.

### Study strengths and limitations

As a strength, this study was conducted based on a nationally representative multi-country dataset that gives a high statistical power to generalize across the region. Additionally, we used a multilevel regression analysis to consider a clustering effect and give unbiased effect size estimates. However, our study has also limitations. First, a cause-and-effect relationship could not be inferred for it was a cross-sectional study design. Second, we could not account for factors like food security status, maternal nutrition during pregnancy for it was a study based on a secondary data analysis. Finally, findings from this study were based on the data obtained from the DHS datasets in the previous decade. Therefore, we recommend other authors to include the most recent data obtained from competent health institutions of countries in the region.

### Policy implications

The double burden of malnutrition, with the coexistence of undernutrition and overweight/obesity, has become a major public health problem in many low-and middle-income countries. Therefore, current national nutrition policies, strategies, and programs need to be tailored for early case identification and management of this concurrent phenomenon. Moreover, one of the most important elements in reaching the Sustainable Development Goals is addressing the double burden of childhood malnutrition. Hence, countries have to move quickly toward a multi-sectoral, all-encompassing strategy that addresses the double burden of malnutrition among children.

## Conclusion

Concurrent stunting and overweight or obesity is becoming an emerging layer of malnutrition. Under five children born in the SSA region had almost a 2% overall risk of developing CSO. This analysis determined that there were regional variations in the burden of CSO in SSA. Age of the children, vaccination status, maternal age, maternal obesity, and region of SSA were significantly associated with under five concurrent stunting and overweight or obesity (CSO). Therefore, nutrition policies and programs should promote quality and nutritious diets to limit the risk of developing CSO in early life. Unvaccinated children, children born to younger and obese women, and those from the West Africa region were most vulnerable.

## Data Availability

The datasets presented in this study are publicly available in online repositories from www.measuredhs.com.

## References

[1] World Health Organization (WHO)., 2022. Fact sheets - Malnutrition [Internet]. [cited 2022 Jan 16]. Available from: https://www.who.int/news-room/fact-sheets/detail/malnutrition.

[2] Calkins K, Devaskar SU (2011). Fetal origins of adult disease. Curr Probl Pediatr Adolesc Health Care.

[3] Organization WH. Guideline: assessing and managing children at primary healthcare facilities to prevent overweight and obesity in the context of the double burden of malnutrition. 2017. 29578661

[4] World Health Organization-double burden of malnutrition., 2022. Double burden of malnutrition [Internet]. World Health Organization; [cited 2022 Jun 2]. Available from: http://www.who.int/nutrition/double-burden-malnutrition/en/.

[5] Chowdhury MRK, Khan HT, Rashid M, Kabir R, Islam S, Islam MS (2021). Differences in risk factors associated with single and multiple concurrent forms of undernutrition (stunting, wasting or underweight) among children under 5 in Bangladesh: a nationally representative cross-sectional study. BMJ open.

[6] Farah AM, Nour TY, Endris BS, Gebreyesus SH (2021). Concurrence of stunting and overweight/obesity among children: evidence from Ethiopia. PLoS ONE.

[7] Atsu BK, Guure C, Laar AK (2017). Determinants of overweight with concurrent stunting among ghanaian children. BMC Pediatr.

[8] Steyn NP, Nel JH (2022). Prevalence and determinants of the double burden of malnutrition with a focus on concurrent stunting and Overweight/Obesity in children and adolescents. Curr Nutr Rep.

[9] Minh Do L, Lissner L, Ascher H (2018). Overweight, stunting, and concurrent overweight and stunting observed over 3 years in vietnamese children. Global Health Action.

[10] Joint UNICEF-, WHO-The World Bank. : Joint child malnutrition Database – Levels and trends. Estimates for 2012. http://www.who.int/nutgrowthdb.

[11] Black RE, Allen LH, Bhutta ZA, Caulfield LE, De Onis M, Ezzati M (2008). Maternal and child undernutrition: global and regional exposures and health consequences. The lancet.

[12] International CSAEaI. Ethiopian Demographic and Health Survey. Central Statistical Agency Addis Ababa, Ethiopia.The DHS Program ICF, Rockville. Maryland, USA,July 2017 [Available from: https://dhsprogram.com/pubs/pdf/FR328/FR328.pdf.

[13] Masibo PK. Trends and determinants of malnutrition among children Age 0–59 months in Kenya (KDHS 1993, 1998, 2003, and 2008-09). ICF International; 2013.

[14] Willey BA, Cameron N, Norris SA, Pettifor JM, Griffiths PL (2009). Socio-economic predictors of stunting in preschool children-a population-based study from Johannesburg and Soweto. South Afr Med J.

[15] Quamme SH, Iversen PO. Prevalence of child stunting in Sub-Saharan Africa and its risk factors. Clin Nutr Open Sci 2022 Apr 1;42:49–61.

[16] Victora CG, Adair L, Fall C, Hallal PC, Martorell R, Richter L (2008). Maternal and child undernutrition: consequences for adult health and human capital. The lancet.

[17] Seidell JC, Halberstadt J (2015). The global burden of obesity and the challenges of prevention. Annals of Nutrition and Metabolism.

[18] Ayele BA, Tiruneh SA, Ayele AA, Zemene MA, Chanie ES, Hailemeskel HS (2022). Prevalence and determinants of overweight/obesity among under-five children in sub-saharan Africa: a multilevel analysis. BMC Pediatr.

[19] Gurnani M, Birken C, Hamilton J (2015). Childhood obesity: causes, consequences, and management. Pediatr Clin.

[20] Fongar A, Gödecke T, Qaim M (2019). Various forms of double burden of malnutrition problems exist in rural Kenya. BMC Public Health.

[21] Fernald LC, Neufeld LM (2007). Overweight with concurrent stunting in very young children from rural Mexico: prevalence and associated factors. Eur J Clin Nutr.

[22] Said-Mohamed R, Allirot X, Sobgui M, Pasquet P (2009). Determinants of overweight associated with stunting in preschool children of Yaounde. Cameroon Annals of human biology.

[23] Rachmi CN, Agho KE, Li M, Baur LA (2016). Stunting coexisting with overweight in 2· 0–4· 9-year-old indonesian children: prevalence, trends and associated risk factors from repeated cross-sectional surveys. Public Health Nutr.

[24] Onis Md, Onyango AW, Borghi E, Siyam A, Nishida C, Siekmann J (2007). Development of a WHO growth reference for school-aged children and adolescents. Bull World Health Organ.

[25] Popkin BM, Corvalan C, Grummer-Strawn LM (2020). Dynamics of the double burden of malnutrition and the changing nutrition reality. The Lancet.

[26] Nel JH, Steyn NP. The Nutrition Transition and the double burden of Malnutrition in Sub-Saharan African Countries: how do these countries compare with the recommended LANCET COMMISSION Global Diet? Int J Environ Res Public Health. 2022;19(24). 10.3390/ijerph192416791 PMC9779835 36554669

[27] Ramirez-Zea M, Kroker-Lobos MF, Close-Fernandez R, Kanter R (2014). The double burden of malnutrition in indigenous and nonindigenous guatemalan populations. Am J Clin Nutr.

[28] Ghattas H, Acharya Y, Jamaluddine Z, Assi M, El Asmar K, Jones AD (2020). Child-level double burden of malnutrition in the MENA and LAC regions: prevalence and social determinants. Matern Child Nutr.

[29] Zhang Y-Q, Li H, Wu H-H, Zong X-N (2021). Stunting, wasting, overweight and their coexistence among children under 7 years in the context of the social rapidly developing: findings from a population-based survey in nine cities of China in 2016. PLoS ONE.

[30] Wells JC, Sawaya AL, Wibaek R, Mwangome M, Poullas MS, Yajnik CS (2020). The double burden of malnutrition: aetiological pathways and consequences for health. The Lancet.

[31] Smith LC, Ruel MT, Ndiaye A (2005). Why is child malnutrition lower in urban than in rural areas? Evidence from 36 developing countries. World Dev.

[32] Goudet SM, Faiz S, Bogin BA, Griffiths PL. Pregnant women’s and community health workers’ perceptions of root causes of malnutrition among infants and young children in the slums of Dhaka, Bangladesh. American Public Health Association; 2011. pp. 1225–33. 10.2105/AJPH.2010.300090 PMC3110238 21653248

[33] de Araújo Morais AH, de Souza Aquino J, da Silva-Maia JK, de Lima Vale SH, Maciel BLL, Passos TS (2021). Nutritional status, diet and viral respiratory infections: perspectives for severe acute respiratory syndrome coronavirus 2. Br J Nutr.

[34] Schaible UE, Kaufmann SHE (2007). Malnutrition and infection: complex mechanisms and global impacts. PLoS Med.

[35] Painter SD, Ovsyannikova IG, Poland GA (2015). The weight of obesity on the human immune response to vaccination. Vaccine.

[36] Kimani-Murage EW (2013). Exploring the paradox: double burden of malnutrition in rural South Africa. Global health action.

[37] Tenenbaum-Gavish K, Hod M (2013). Impact of maternal obesity on fetal health. Fetal Diagn Ther.

[38] Wojcicki JM (2014). The double burden household in sub-saharan Africa: maternal overweight and obesity and childhood undernutrition from the year 2000: results from World Health Organization Data (WHO) and demographic health surveys (DHS). BMC Public Health.

[39] Whitaker RC, Wright JA, Pepe MS, Seidel KD, Dietz WH (1997). Predicting obesity in young adulthood from childhood and parental obesity. N Engl J Med.

[40] UNICEF / WHO / World Bank Group, UNICEF, WHO WBG., Joint Child Malnutrition Estimates Key Findings. Unichef. 2021. availible at: https://www.who.int/news/item/06-05-2021-the-unicef-who-wb-joint-child-malnutrition-estimates-group-released-new-data-for-2021.

